# Medium-Level Laser in Chronic Tinnitus Treatment

**DOI:** 10.1155/2013/324234

**Published:** 2013-11-05

**Authors:** K. Dejakum, J. Piegger, C. Plewka, A. Gunkel, W. Thumfart, S. Kudaibergenova, G. Goebel, F. Kral, W. Freysinger

**Affiliations:** ^1^ENT Department, Regional Hospital, Endach 27, 6330 Kufstein, Austria; ^2^Pradlerstraße 4, 6020 Innsbruck, Austria; ^3^University ENT Hospital, Paracelsus Private Medical University, Müllner Hauptstraße 48, 5020 Salzburg, Austria; ^4^University Hospital of Otorhinolaryngology, Innsbruck Medical University, Anichstraße 35, 6020 Innsbruck, Austria; ^5^ENT Department, S.D. Asfendiyarov Kazakh National Medical University, Almaty 050012, Kazakhstan; ^6^Department of Medical Statistics, Informatics and Health Economics, Innsbruck Medical University, 6020 Innsbruck, Austria

## Abstract

The purpose of this study was to evaluate the effect of medium-level laser therapy in chronic tinnitus treatment. In a prospective double-blind placebo-controlled trial, either active laser (450 mW, 830 nm combined Ga-Al-As diode laser) or placebo irradiation was applied through the external acoustic meatus of the affected ear towards the cochlea. Fourty-eight patients with chronic tinnitus were studied. The main outcome was measured using the Goebel tinnitus questionnaire, visual analogue scales measuring the perceived loudness of tinnitus, the annoyance associated with tinnitus, and the degree of attention paid to tinnitus as well as psycho-acoustical matches of tinnitus pitch and loudness. The results did show only very moderate temporary improvement of tinnitus. Moreover, no statistically relevant differences between laser and placebo group could be found. We conclude that medium-level laser therapy cannot be regarded as an effective treatment of chronic tinnitus in our therapy regime considering the limited number of patients included in our study.

## 1. Introduction

The treatment of chronic tinnitus often leads to frustration both on the side of the patient and the therapist. This is partly due to the wide range of possible aetiologies including cochlear damage, myoclonic/myogelosis problems, and central nervous system pathologies [1–6]. During the last decade, however, the concept of “Tinnitus Retraining Therapy” has brought at least some relief for those who suffer most [7, 8]. Also cognitive behavioural treatments are efficient [9, 10]. The lack of an efficient medical or surgical cure has prompted researchers to seek novel treatments though. Some of the more sophisticated new treatments are hyperbaric oxygen therapy [11], transcranial magnetic stimulation [12], botulinum toxin treatment of essential palatal myoclonus tinnitus [13], and GABAA: benzodiazepine chloride receptor-targeted therapy [14].

Another approach is the low/medium-level laser therapy. At the end of the last century. low-level lasers (with about 50 mW power), which had been successful in treatment of wound healing and pain [15, 16], have been used on tinnitus patients, assuming an athermic stimulation of biochemical processes in the inner ear induced by light [17]. Conflicting studies have been published ranging from success rates over 75% [18–20] to no significant improvement at all [21–25]. 

In recent years health professionals went on to use laser devices with medium power (about 450 mW) and enhanced both time and frequency of laser exposition aiming at depositing a total laser energy of 30 to 80 times compared to the earlier studies. For those settings there are no research data at all, to the best of our knowledge (as of July 2005). Specifically, cerebral effects of laser irradiation on the cochlea could be quantitatively determined in fMRI [26] and in a recent animal study [27]. It is the aim of this study to evaluate the effect of medium-level laser therapy (MLLT) in chronic tinnitus in a randomized, placebo-controlled double-blind design. 

## 2. Materials and Methods

### 2.1. Subjects

Forty-eight patients (23 women, 25 men) suffering from chronic tinnitus (history more than 6 months) were recruited consecutively at the Ear, Nose, and Throat Department of the Innsbruck Medical University between June, 2002 and March, 2004 and were randomly assigned to either therapy group A or B. Patients' age ranged from 16 to 70 years at entry (average of 50.4 years). Other than tinnitus and sensoneurinal hearing loss all participants were healthy and were not receiving any other tinnitus treatment—although many patients had received other therapies before (including prednisolone/pentoxifylline infusions, hyperbaric oxygen therapy, and tinnitus retraining therapy). One patient quit after two sessions and was excluded from the statistical analysis.

The routine tinnitus patient screening with MR imaging excluded other origins of tinnitus, such as acoustic neurinoma or cerebral tumors or cerebral vessel malformations.

### 2.2. Pre- and Posttreatment Evaluations

In this placebo-controlled double-blind setting, 22 subjects were randomly allocated to the active laser treatment (group B) and 25 to a placebo treatment (group A). Before laser therapy the subjects underwent an ENT examination, an audiometric assessment (including pure tone audiometry, stapedial reflexes, middle ear pressure measurement as well as tinnitus pitch and loudness matches), and an assessment of the severity of tinnitus by means of the Goebel tinnitus questionnaire [28] and visual analogue scales (VAS) measuring the perceived loudness of tinnitus, the annoyance associated with tinnitus and the degree of attention paid to tinnitus [25]. The scales each ranged from 1 to 10 (1 = no disturbance, 10 = complete disturbance). Furthermore, blood tests were done (total cholesterol, HDL, LDL and triglycerides) to examine a hypothetic connection between laser treatment and cholesterol levels. The same examinations were repeated at the end of treatment. Six weeks after therapy only the Goebel questionnaire and the VAS were repeated. The show-up rate at the control examination was 98%. There was no longer followup in this study. 

### 2.3. Treatment Setting

**Figure 1:**
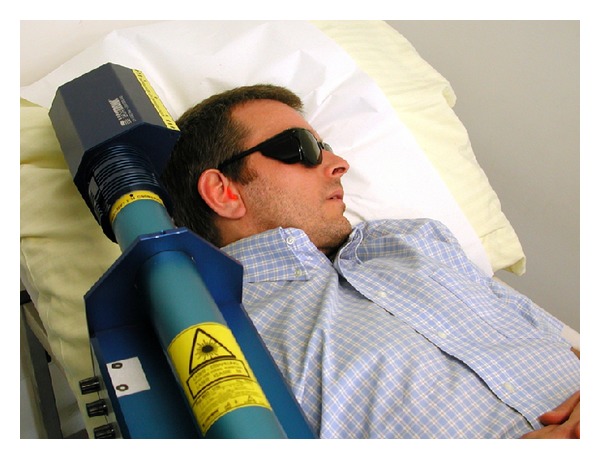
Treatment setting.

The laser treatment consisted of twelve 30-minute sessions over a period of four weeks (three times a week). The patients were treated in a quiet environment (Figure 1). They could listen to relaxing background music to mask the beeping noise of the laser device. During laser exposure, the subjects were lying supinely on a couch with a pillow, wearing a pair of laser protective goggles (Figure 1), which were also used by the attending physician. The laser-emitting area of the device was placed at a distance of 15 cm to the ear. Only the affected ear was treated. If patients had bilateral tinnitus, the side with the higher tinnitus loudness match was treated. If patients could not spatially allocate their tinnitus, the study protocol was such as to treat the right ear by default.

### 2.4. Laser Equipment

**Figure 2:**
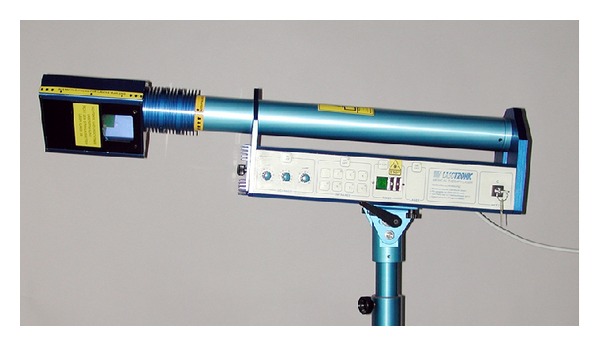
Lasotronic Laser device: the device can be adjusted in height, and the laser head can be tilted and rotated to reach the correct position of the laser beam. The turning knobs on the left serve to adjust focus and irradiation patterns. The key on the right is the main power switch.

We used two identically looking laser devices (Figure 2) produced by Lasotronic (Hengersberg/Germany). One had an active combined gallium aluminium arsenide diode laser with a wavelength of 830 nm (infrared radiation) and maximum output power of 450 mW. In the other, the infrared laser was deactivated. Additionally, both devices had a red light laser pointer (630 nm, <1 mW output) used as an aiming beam to find the correct position for irradiation. Without technical means the involved staff could not determine which unit was the placebo one. We used the transmeatal approach (the beam was aimed at the acoustic meatus towards the tympanic membrane) as in some studies before [18, 20, 22, 25]. Other researchers preferred the transtemporal approach [19, 23], but we found out in an earlier laboratory experiment that the transmeatal approach yields higher light intensities (although within the order of magnitude of nW) medially to the inner ear [29]. The probes were randomly coded (groups A and B) by a technician not involved in the study to ensure the double-blind design. The total laser energy applied to each patient amounted to 9.700 J. The laser power was measured in regular intervals (biweekly by the technician) to guarantee the constancy of delivered power. 

The Med 1000 device used is a CE-0297-certified device and fulfils the according laser safety regulations as of § 22 (1) European Union Medical Device Directive (2002), current at the time the study was initiated. These regulations are now replaced by the medical device regulation of the European Union.

### 2.5. Statistical Analysis

For statistical analysis we used SPSS 10.0 (Chicago, Ill., USA). The data analysed were total tinnitus score (Goebel and Hiller [28]) as well as the subdivisions of psychological strain, penetrance of tinnitus, hearing problems, sleeping impairment, and somatic impact.

Furthermore, loudness, annoyance, attention, sleeping disorders, and somatic complaints were measured with the visual analogue scale (VAS). 5 dB differences of tinnitus loudness match, and 10 dB hearing differences were monitored in pure-tone audiometry. The HDL/LDL ratio was measured on blood samples of the volunteers. The data were collected for both groups at the start and the end of treatment. The Goebel VAS was filled in again six weeks after treatment end.

Descriptive data are shown as mean (+/− statistical deviation) for parametric data or median (25% percentile/75% percentile) for nonparametric data. For comparisons between groups we used the *t*-Test or Mann-Witney *U* Test (Wilcoxon Test, Kruskal-Wallis Test) as the appropriate statistical tests. A *P*-value of 0.05 was considered statistically significant. 

## 3. Results

To visualize our results we used box plots: the columns show the inner 50% of measurements, the horizontal bars within the columns show the median. Within the upper and lower horizontal bars lie all measurements. The first column (red) shows data taken at the beginning of treatment, the second one (yellow) shows data taken at the end of treatment, and the third (blue) shows data taken 6 weeks after the end of treatment. The *x*-axis shows the number of patients in each group, the *y*-axis shows the measurements taken.

**Figure 3:**
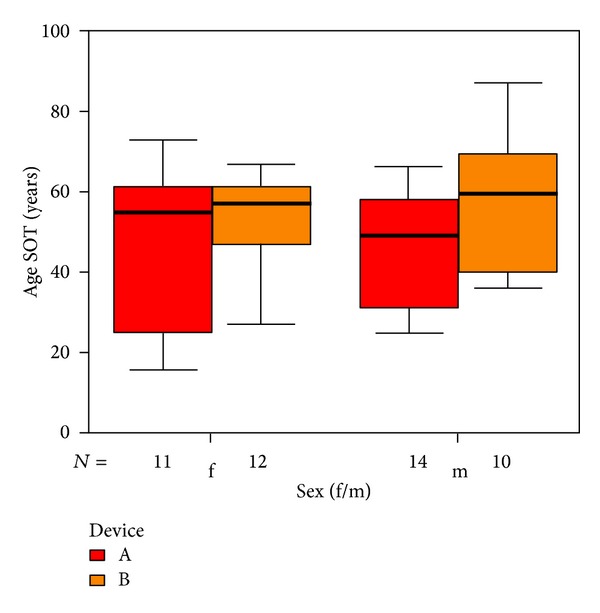
Distribution of age and sex with respect to devices A and B.

The average age of the 47 subjects (23 women, 24 men) completing the study was 50.4 years (range: 16–70 years). The distribution of the groups and devices are shown in Figure 3. 

**Figure 4:**
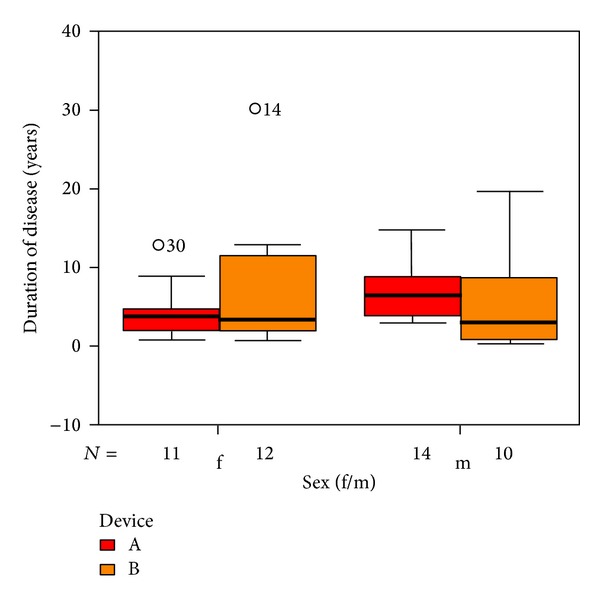
Distribution of duration of tinnitus and sex with respect to devices A and B. The patients indicated with “*⚪*” are statistical outliers.

Duration of tinnitus ranged from 6 months to 20 years (median 4.5 years) as shown in Figure 4.

### 3.1. Audiological Assessment

No statistically significant difference was found in pure tone audiometry before and after treatment in the laser and the placebo groups. A difference in pure tone audiometry was defined as difference of at least 10 dB in at least 3 frequencies between 125 Hz and 8 kHz. Smaller differences are most certainly due to measurement uncertainties.

### 3.2. Assessment of Treatment Outcome

**Figure 5:**
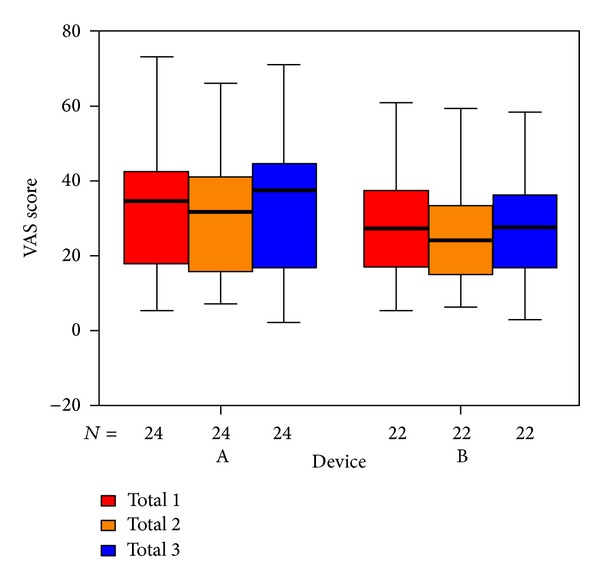
Goebel total tinnitus score at start of treatment (SOT), end of treatment (EOT), and control (CTR) with respect to devices A and B of the associated number of patients.

Our main assessment tool was the Goebel tinnitus questionnaire [28], which was filled out by the volunteers at the beginning and the end of treatment as well as 6 weeks after treatment (backflow rate of 98%). Overall, patients experienced a moderate subjective improvement at the end of treatment (Figure 5) both in the laser and placebo group, which is consistent with other tinnitus studies [25, 30]. Six weeks after therapy this effect was reversed. At this time, both groups showed a moderate increase of the overall score. Both findings were not statistically significant, yet. 

**Figure 6:**
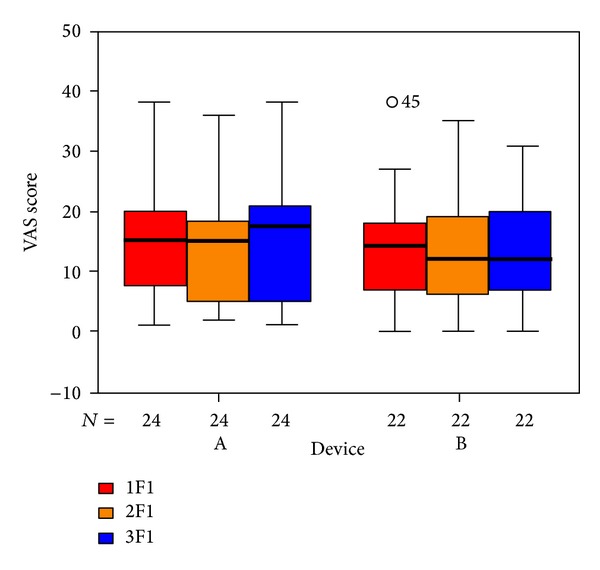
Goebel psychological strain subdivision tinnitus score at SOT (1F1, red), EOT (2F1, orange), and CTR (3F1, blue) with respect to devices A and B of the associated number of patients. The patients indicated with “*⚪*” are statistical outliers.

**Figure 7:**
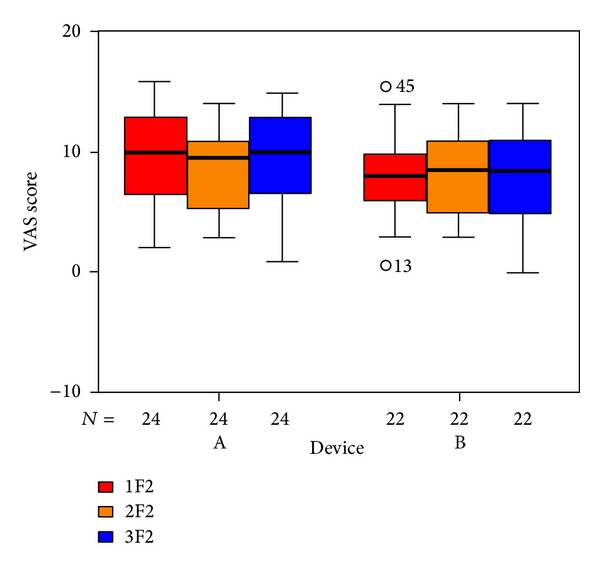
Goebel penetrance of tinnitus subdivision and tinnitus score at SOT (1F2, red), EOT (2F2, orange), and CTR (3F2, blue) with respect to devices A and B of the associated number of patients. The patients indicated with “*⚪*” are statistical outliers.

**Figure 8:**
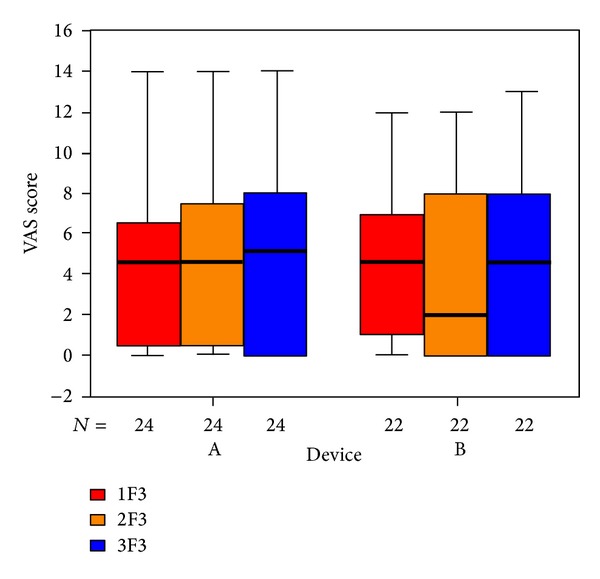
Goebel hearing problems subdivision and tinnitus score at SOT (1F3, red), EOT (2F3, orange), and CTR (3F3, blue) with respect to devices A and B of the associated number of patients.

**Figure 9:**
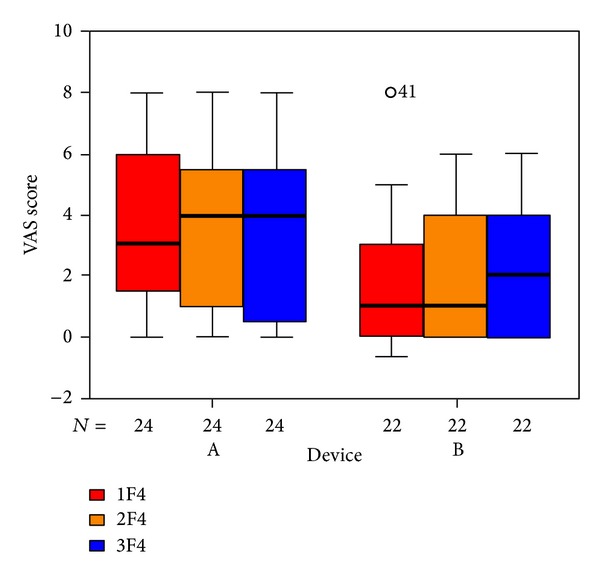
Goebel sleeping disorders subdivision and tinnitus score at SOT (1F4, red), EOT (2F4, orange), and CTR (3F4, blue) with respect to devices A and B of the associated number of patients. The patients indicated with “*⚪*” are statistical outliers.

**Figure 10:**
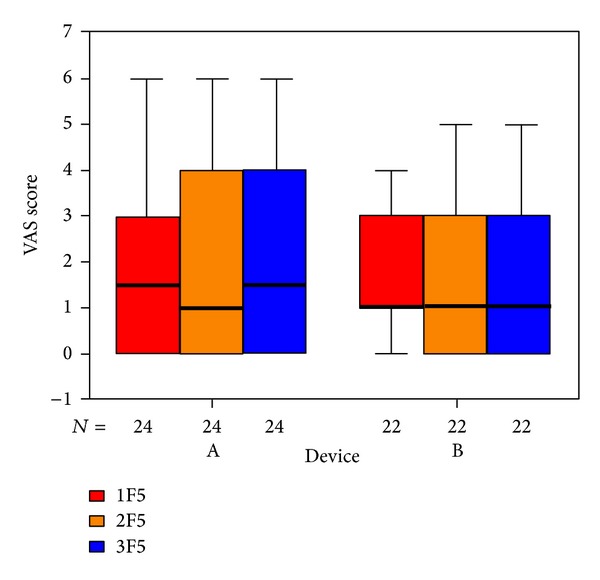
Goebel somatic complaints subdivision tinnitus score at SOT (1F51, red), EOT (2F5, orange), and CTR (3F5, blue) with respect to devices A and B of the associated number of patients.

The items in the questionnaire are assigned to five subdivisions. The outcome for those questions related to psychological stress (Figure 6), penetrance of tinnitus (Figure 7), hearing problems (Figure 8), sleeping disorders (Figure 9), and somatic complaints (Figure 10), respectively, is listed below. 

Subjects in the laser group had a slight improvement in hearing problems (Figure 8) at the end of therapy (again not statistically significant). The effect was not found at the 6-week control. Furthermore there was no difference in pure tone audiometry. No differences were found in the other subdivisions of the Goebel questionnaire.

**Figure 11:**
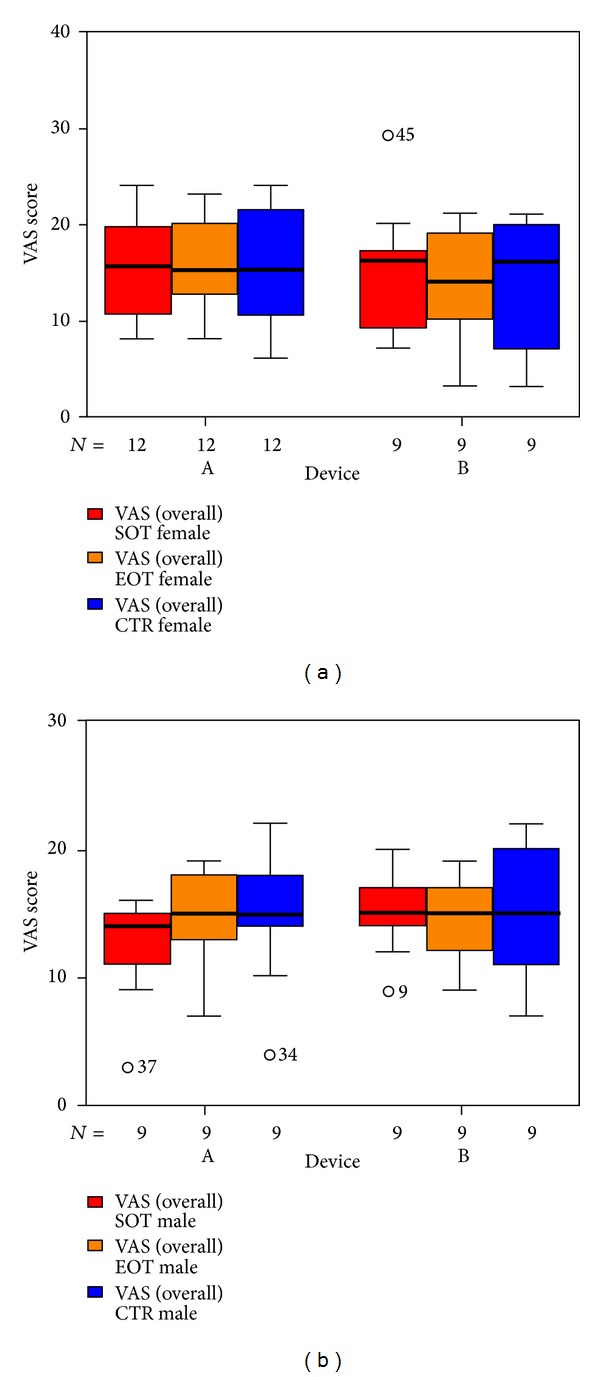
VAS total at SOT, EOT, and CTR with respect to devices A and B, for male and female volunteers, respectively. The patients indicated with “*⚪*” are statistical outliers.

**Figure 12:**
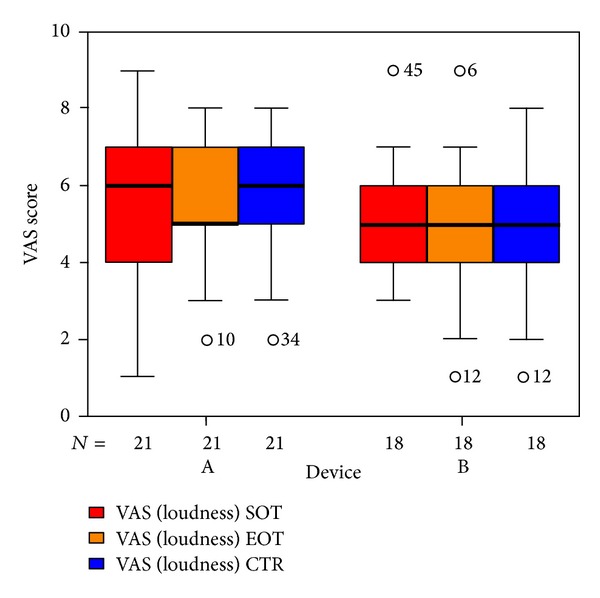
VAS loudness of tinnitus at SOT, EOT, and CTR with respect to devices A and B (not differentiated against sex). The patients indicated with “*⚪*” are statistical outliers.

**Figure 13:**
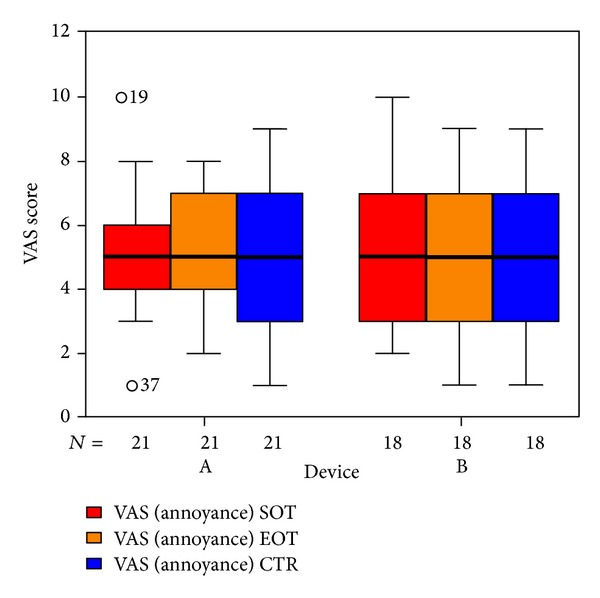
VAS annoyance associated with tinnitus at SOT, EOT, and CTR with respect to devices A and B (not differentiated against sex). The patients indicated with “*⚪*” are statistical outliers.

**Figure 14:**
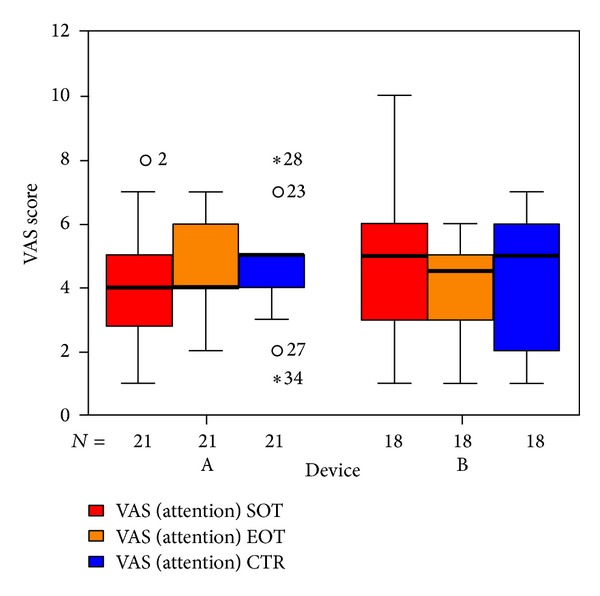
VAS degree of attention paid to tinnitus at SOT, EOT, and CTR with respect to devices A and B (not differentiated against sex). The patients indicated with “∗” or “*⚪*” are statistical outliers.

We used three VAS measuring the perceived loudness of tinnitus, the annoyance associated with tinnitus, and the degree of attention paid to tinnitus. There were no significant differences in total VAS (Figure 11). Tinnitus loudness (Figure 12) decreased in the placebo group at the end of treatment (not statistically significant); six weeks after therapy it was the same as before. The median of the annoyance-score associated with tinnitus (Figure 13) increased in group B (laser group) at the 6-week control (again not statistically significant). There were no changes in the degree of attention paid to tinnitus (Figure 14). 

**Figure 15:**
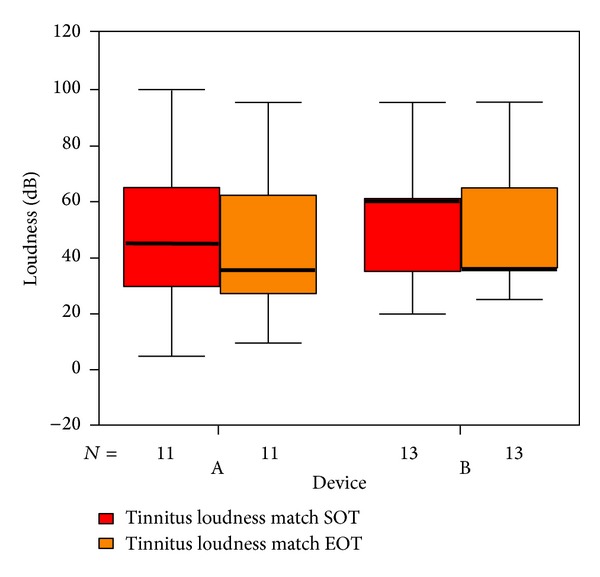
Tinnitus loudness match at SOT and EOT with respect to devices A and B (not differentiated against sex).

**Figure 16:**
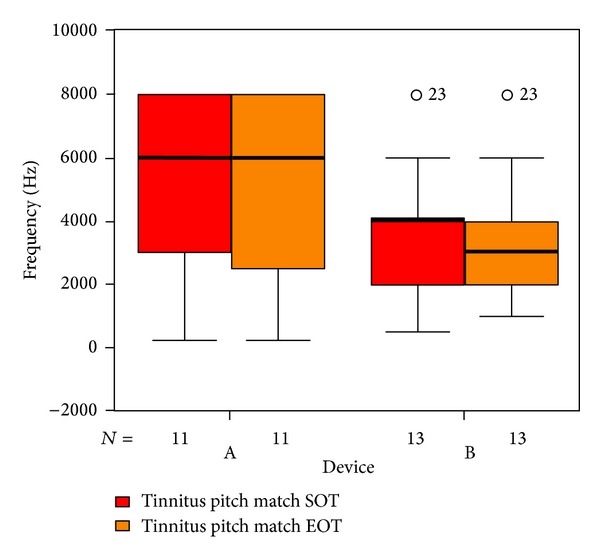
Tinnitus pitch match at SOT and EOT with respect to devices A and B (not differentiated against sex). The patients indicated with “*⚪*” are statistical outliers.

Tinnitus loudness matches (Figure 15) show a median improvement of 10 dB in the placebo group against no differences in the laser group. Tinnitus pitch matches (Figure 16) were generally higher in the placebo group (median of 6000 Hz in group A versus 4000 Hz in group B). At the end of therapy, the pitch matches in the laser group were down to 3000 Hz against no differences in the placebo group (statistically not significant).

We could not find any statistically significant changes in total cholesterol, LDL/HDL ratio, and triglycerides in either group A or B.

## 4. Discussion

Since the emergence of the low-level laser therapy for tinnitus in the late 1980s only a few reports of its effectiveness have been published. Most of them combined laser with Ginkgo Biloba, an acclaimed vasodilatator. One uncontrolled study reported improvement of tinnitus in 75% and improvement in hearing in 80% of subjects [18]. Two other uncontrolled trials still found tinnitus relief in 55% [19] and 25% [21], respectively. One controlled single-blind study showed beneficial effects in 50% of the active treatment group compared to 5% in the placebo group [22]. Two other studies (one single-blind [23] and one double-blind [25]) could not find any statistical significant differences between active treatment and placebo groups. The treatment methods in all these studies were similar, He-Ne and/or Ga-Al-As Lasers with wavelengths between 630 and 900 nm and maximum output power between 10 and 50 mW have been pointed at the mastoid or the external acoustic meatus. The bias inherent to single-blind and especially to uncontrolled studies has limited the value of many of these trials and compromised their conclusions.

In our placebo-controlled, double-blind study a very moderate improvement of tinnitus was found in the laser and the placebo group at the end of treatment according to the Goebel tinnitus questionnaire. We think this was mainly due to the placebo effect [30]. Patients were cared for; they had someone to share their sorrows with. At the control examination six weeks after therapy the effect was lost. In other studies, the placebo effect seems to be of much more relevance [19, 23, 25]. The reason for this difference may be that in other studies patients were highly motivated and expected a quick recovery, whereas we tried to inform our patients in a rather neutral way about the prospects of the treatment and the study design. They also knew that the chance to get a placebo treatment was 50%. However, we did not find an increase in attention paid to tinnitus as in one similar study [25], which might happen because the subjects have to spend more time thinking about the annoying sensations during treatment as well as before and after therapy. The only other change observed was a moderate increase in tinnitus annoyance in the laser group at the 6-week control. We have no explanation for this besides the lacking statistical significance. 

The present results are in line with current work on treating tinnitus with low-level laser radiation [27]. The work of Okhovat et al. [31] is a self-controlled and no double-blinded clinical study. As such it is not comparable to our study. However, the results found are in line with ours. The work of [32] is fully in line with our results. The work of Cuda and de Caria [33] refers to treating tinnitus with a combination of hypnotherapeutic and muscle-relactant techniques and thus is hardly comparable to the present study. In [34] a positive influence of LLLT is reported without statistical significance, and [35] includes patients with Ménière's disease and is not run as a double-blind randomized study.

Our statistical analysis would undoubtedly have been more conclusive if we had included much more patients, which we did not due to the very time consuming treatment setting. According to our statistical data, a patient collective of 1000 subjects would be adequate and thus beyond our possibilities. However, under the given conditions we could not find any significant positive or negative effect of medium-level laser therapy on chronic tinnitus.

## 5. Conclusion

We conclude that the increase of the deposited total laser energy (up to a factor of 80), which forms the only difference between low-level laser therapy and medium-level laser therapy does not result in a statistically significant reduction of symptoms in chronic tinnitus.
